# Uterine Displacements

**Published:** 1870-04

**Authors:** L. A. Babcock

**Affiliations:** Freeport, Ill.


### UTERINE DISPLACEMENTS.

BY L. A. BABCOCK, M. D., FREEPORT, ILL.

Displacements of the uterus are of three kinds, viz: PROLAPSUS, RETROVERSION and ANTEVERSION. The uterus occupies, normally, very nearly a central position in the pelvis, being perhaps a little nearer to the sacrum than to the pubis, its long axis should stand at about right angles to that of the vagina, the fundus pointing in the direction of the umbilicus, and the os towards the end of the coccyx. Prolapsus signifies a sinking of the uterus below its normal position, and sometimes it sinks nearly or quite down to the os externum; when it protrudes beyond the vulva, it is called procidedtia uteri. The fundus may be tilted a little one way or the other without the position being necessarily abnormal. The condition and contents of the bladder and rectum may temporarily influence it to some extent.

If it turns forward or backwards for 25 degrees or 30 degrees, it does not amount to malposition; but if to 40 degrees in either direction, without soon rectifying itself, it is abnormal, and usually goes from bad to worse, till the malposition becomes persistent. If the uterus falls backward in a line drawn from the os to the promontory of the sacrum, it will describe an angle of 45 degrees, and it will present its broadest surface to the pressure of the superincumbent vicera, which will necessarily force it eventually lower; and if turned forward to the same extent, the same power exerted on its broad posterior surface necessarily increases this abnormal tendency. But an anteversion never goes relatively to so great an extent as a retroversion, simply because it meets with more resistance. Anteversion often stops at 45 degrees, but may go to 90 degrees, as when we have a complete version, with the whole organ lying flatly down on the entire wall of the vagina, and parallel with it, while a retroversion seldom or never stops under 90 degrees, simply because there is less opposition to its downward progress. It then follows that if the fundus of the uterus is found constantly lying just behind or even near the symphisis pubis, it is anteversion; but if it is found lying persistently back under the promontory of the sacrum, it is a retroversion.

I purpose in this article to collect the best information regarding the pathological anatomy of prolapse of the uterus, without aiming so much at originality as correct statement of facts, gleaning from the best authors and clinical works without credit or comment.

By falling of the womb, we generally understand a downward displacement of the organ, in the direction of its longitudinal axis, consequently corresponding with the axis of the pelvis.

The anatomical relations between the uterus and vagina will not allow the assumption of a sinking of the former, either without a corresponding shortening of the vagina by a kind of shrinking, which shortening, owing to the external attachment of the vagina, cannot easily take place without the latter being *inverted* by the sinking uterus. Hence, in most cases, *prolapsus uteri* is combined with *inversion of the vagina*, and according as this condition varies, degrees of prolapsus have been classified as "discensus uteri," in which the uppermost portion of the vagina is inverted. 2d. *Incomplete prolapsus uteri*, causing inversion of more than one-half of the vagina. And 3d. When the uterus has descended as low as the vagina will allow, the whole length of which has been inverted, constituting procidentia uteri, (hysterocele.)

According to the degree, various accessory conditions and consequences are developed. But in any well marked case of prolapsus there is tumefaction of the uterus, and hypertrophy of the vagina, which, in well pronounced cases, that have existed a long time, scarcely exhibit a trace of the transverse folds which the vaginal mucus membrane possesses, in its normal condition. And, notwithstanding the considerable stretching of the vagina in its tranverse diameter, its walls are always much thickened, owing to the proliferation of its epethelium, which forming successive layers, may attain the thickness of a line, and appear in the dead body in the form of shreds. The mucus membrane underlying it is thicker than usual. The submucous *areolar* tissue more resistant and more or less œdematious, and the muscular coat of the vagina is always considerably increased in substance.

If we open the abdominal cavity in cases of complete prolapsus, we will always find, between the bladder and rectum, a funnel-shaped inversion of the peritoneum towards the floor of the pelvis, at either side of the upper and lower entrance of this excavation, the ovaries and oviducts are found drawn towards its margin, and frequently lying somewhat anteriorly, and in the depths of the inversion the fundus uteri will be discovered. The broad ligaments, especially in the begining of prolapsus, are always in a state of considerable *tension*, and are sometimes stretched in the shape of folds, which ascend obliquely from the lower part of the inversion towards each side. The impeded reflux of venous blood occasioned thereby, is apparent in the uterus, ovaries and oviducts, as also in the everted vagina, presenting the appearance of passive hyperæmia, and even stagnation from the intense varicose distention of the veins. The uterus becomes larger and longer, its walls are moister, and in recent cases softened and relaxed. The mucous membrane is always in a state of hypersecretion and catarrh, and sometimes the cavity of body and fundus is distended by mucous, especially if the elongation of the organ be combined with a stricture of the internal orifice. The relaxation of the uterine tissue is noticeable in the region of the external orifice, and consequently, in what was previously the vaginial portion and lower segment of the cervix, which part often assumes a spongy softness. This relaxation is owing to the varicose condition of the blood vessels, and absorption of the cervical tissues.

The intimate connection of the bladder and rectum with the vagina, will not allow to suppose a complete inversion of the latter, without *traction* of the connecting interstitial tissue, and consequently also of the posterior wall of the bladder, and anterior one of the rectum. In consequence of these conditions, parts of the neighboring organs are drawn down into the everted vagina.

No Physician of experience needs a treatise on the causes of prolapsus uteri, nor the reason of its increased prevalence; the relaxation and tenuity of the ligaments, hydrops, *ascites*, encysted exudations, pseudo-membranous–adhesions, ulcers and catarrh, tells too plainly of violations of physical law in periods of menstruation. It hints too strongly of Periodical drops and abortive nostrums to prevent yearly increase in families. Tight lacing, forcing the bowels down into the pelvic cavity, and exposure of the limbs to sudden extremes of cold and heat; the constant use of stringent and caustic infections, (which in my way of thinking is the worst practice any woman was ever guilty of) all tend to bring on hypertrophy and prolapsus uteri.

The profession cannot correct the evils of society nor stop the degrading custom of habitual use of drugs, to counteract physical laws. We have to deal with facts as they exist, and diagnose a case from its history and present condition, without looking back to our grandmothers to see what diseases they may or may not have had in a former age of prudence, purity and virtue. Our grandmothers may have been poor in this world's goods, but they were rich in all the physical qualities of perfect, healthy women.

There is, perhaps, but little if any difference of opinion in the profession, as to the general pathology in prolapsus uteri. The diagnosis is easy, and but little chance of error in discrimination of this displacement from any other disease.

But when we come to consider the therapeutics, which is the most important part of this subject, being in fact, the great end of both the principles and practice of medicine, so many conflicting opinions prevail, both in this country and Europe, that one can hardly speak frankly on the subject, without a fear that he may hurt the feelings of some noted author who has written a book on the subject.

In a long practice of nearly forty years, I have collected in this country and in Europe, a large pile of Instruments that have been invented and used in the treatment of prolapsus uteri. And now as I look them over, I see all shapes, sizes and materials existing in the mineral and vegitable universe. It is needless to name them over, as all most all seem to have been invented and brought into use with an utter disregard of the pathological fact before stated, to wit: *The relaxation of the ligaments, expansion and inversion of the vagina, and traction and descent of the posterior wall of the bladder, and the anterior of the rectum.*

If a physician is called to treat a dislocation, he proceeds at once to reduce the displacement by putting the bone into its proper place, and if there is considerable laceration of the soft parts, he directs cold applications, and perhaps a sling, leaving nature to do its own legitimate work.

We never think of applying astringent lotions of Nit. Arg'en. to the projection of the external condyle of the humerus in dislocations of the elbow. And why not treat a displacement of the uterus as practically and as simply as we treat displacement of a bone?

What can be more humiliating to the profession than a historical review of the means used in the treatment of prolapsus, glass globes, balls, tin boxes, wooden buttons, rubber wind-bags, horse shoes, steel springs, spiral springs, sponge tents, linen and cotton rags; and recently I took out of the vagina of a patient that came to me for examamination, a crash towel.

A Homeopathic Doctor across the way, has a mode of treating his patients, which is very nice and comfortable. He uses round balls of mutton tallow, the size of bird's eggs, one of which he introduces every day, (except perhaps three in each month,) for years, if he can keep the patient.

A very celebrated professor of Obstetrics recommends a walk of a mile in the open air every morning. And others of no less note—confinement to bed, and daily visits for six months or a year—making it a paying business to keep such patients on hand as long as their money lasts.

To treat successfully prolapsus uteri, the womb must be restored to its normal position in the pelvis; and then it must be held there by an instrument that does not irritate the crevix or expand the vagina transversely, nor rest on the perineum for its support. The inversion of the vagina must be overcome, and the walls of the bladder and rectum relieved of the downward traction by the contraction of the expanded vagina into its normal condition.

It will be seen at once that this cannot be accomplished by any instrument that rests on the vaginal walls for support. The instrument must be supported externally, and not interfere with, or prevent the contraction of the vagina. The instrument must not interfere either, with the bowel or urethra, and should not be large or burdensome to use. It should not be made of any material that corrodes or increases the relaxation and catarrh. I am fully of the opinion that rubber in any form acts as an irritant; and in proof of this, I will state that I have never met with a lady who wore teeth set in rubber for a number of years, who did not have follicular irritation of the mouth and throat.

I am very decidedly in favor of the use of pure polished silver in making an instrument to support the womb in prolapsus, because you can have the instrument light and thin, and yet strong enough to hold up the uterus by a stem not larger than a goose quill, which will not interfere with the vagina, rectum or canal of the bladder. The cup must be made in the exact shape of the cervix, and silver can be worked down to the thickness of two lines, and yet be held strongly on the stem, with a perfectly smooth polished surface, so that no irritation will be caused.

More than twenty years ago I had one made for a patient of mine, which proved to be successful; but the making of it cost me over $50, which was too expensive for most patients. I tried to make it cheaper and more practicable, and after long study and many experiments, I have made it so simple and practical, that nearly two thousand Physicians, who have used them, assure me of its complete success in the treatment of prolapsus uteri.

Another reason why I prefer to use pure silver is, that by putting a small piece of zinc into the hollow tube, into which the handle enters, a feeble current of electricity is produced by the action of the urine on the handle, in conjunction with the copper plate in the belt, keeping up constant contraction of muscles and ligaments, reducing the hypertrophy of vagania and uterus quicker than by any other means in my experience; and the zinc can be removed any moment the action is no longer needed.

Ulcers and inflamations of course, demand appropriate treatment before the instrument can be used. But I believe the old treatment may be improved and made as practical and salutary, as the improved treatment of retropharyngeal abscess, or diptheritic exudative inflamation of the pharynx.

---

A lunatic at Seymour, Ind., put one end of an old gun-barrel in the fire, and looked down the muzzle with one eye to see if it was loaded. It was the opinion of the parties who scraped the man's brains upon a dust-pan, that the gun was loaded, and the coroner's jury concurred.

